# MiRNA expression profiles in the brains of mice infected with scrapie agents 139A, ME7 and S15

**DOI:** 10.1038/emi.2016.120

**Published:** 2016-11-09

**Authors:** Chen Gao, Jing Wei, Bao-Yun Zhang, Qiang Shi, Cao Chen, Jing Wang, Qi Shi, Xiao-Ping Dong

**Affiliations:** 1 State Key Laboratory for Infectious Disease Prevention and Control, Collaborative Innovation Center for Diagnosis and Treatment of Infectious Diseases (Zhejiang University), Prion Department, National Institute for Viral Disease Control and Prevention, Chinese Center for Disease Control and Prevention, Beijing 102206, China; 2 Chinese Academy of Sciences Key Laboratory of Pathogenic Microbiology and Immunology, Institute of Microbiology, Chinese Academy of Sciences, Beijing 100101, China

**Keywords:** deep sequencing, microRNA, prion, scrapie, TSE

## Abstract

MicroRNA (miRNA) is a class of non-coding endogenous small-molecule single-stranded RNA that regulates complementary mRNA through degradation or translation of the mRNA targets. Usually, miRNAs show remarkable cell and tissues specificity. Recently, alterations in a set of miRNAs in the brains of patients with certain neurodegenerative diseases, including prion diseases, have been reported. In this study, using deep sequencing technology, miRNA expression profiles in the brains of mice infected with scrapie agents 139A, ME7 and S15 at a terminal stage were comparatively analysed. In total, 57, 94 and 135 differentially expressed miRNAs were identified in the pooled brain samples of 139A-, ME7- and S15-infected mice, respectively, compared with the brains of age-matched normal controls. Among them, 22 were commonly increased and 14 were commonly decreased in the brains of all three infected models. In addition, a reduction in the expression of two novel miRNAs was also commonly observed. Quantitative PCR with reverse transcription analysis of six randomly selected commonly increased and decreased miRNAs in the brains of the three infected mouse models, as well as the two novel miRNAs, verified that the expression patterns were comparable to the deep sequencing data. KEGG analysis of the differentially expressed miRNAs revealed the involvement of similar pathways in all three types of infected animals. Comprehensive analysis of these miRNA profiles not only provides useful clues for understanding prion biology but also is beneficial in the search for possible diagnostic marker(s) for prion diseases.

## INTRODUCTION

Prion diseases, or transmissible spongiform encephalopathies (TSEs), are a family of rare progressive neurodegenerative disorders that affect both humans and animals, and include bovine spongiform encephalopathy (BSE) in cattle, scrapie in sheep and goat, and chronic wasting disease of deer and elk, as well as Creutzfeldt–Jakob disease (CJD), Kuru, fatal familial insomnia and Gerstmann–Straussler–Scheinker syndrome in humans. They are distinguished by long incubation periods, characteristic spongiform changes associated with neuronal loss and a failure to induce an inflammatory response. The causative agents of TSEs are believed to be prions.

MicroRNAs (miRNAs) are a class of small, non-coding RNAs that mediate RNA silencing and post-transcriptional regulation of gene expression by binding to the 3′ untranslated region of mRNAs. They are single-stranded RNAs and are usually ~22 nucleotides long. The distribution of miRNAs has been shown to be tissue and cell specific, which is believed to be essential for maintaining cell phenotype through the regulation of intracellular protein mRNA expression profiles.^1^

MiRNA in brain tissue is highly conserved, and brain tissue is extremely rich in miRNA.^2^ Compelling studies have already linked the expression of miRNAs to the control of neuronal development and differentiation. Recently, the influences of miRNAs on neuronal survival and the accumulation of toxic proteins that are associated with neurodegeneration have attracted much attention. In Alzheimer disease (AD), miR-107 is significantly reduced in the brains of AD patients, even in the early stages of the disease. MiR-107 is able to regulate the level of mRNA encoding β-site amyloid precursor protein (APP)-cleaving enzyme1, which is involved in the cleavage of APP to produce Aβ, a critical marker of Alzheimer's disease pathology.^3^ In Parkinson's disease (PD), miR-133b is missing in dopamine neurons.^4^ The polymorphism, rs12720208, associated with PD affects the binding of miR-433 to the regulatory sequences of fibroblast growth factor 20, resulting in increased α-synuclein.^5^ Higher levels of brain miR-7in PD patients have also been detected, which can lead to a reduction in α-synuclein.^6^ In Huntington disease, abnormal miRNA expression that may affect the transcription and translation of disease-associated genes has been observed.^7^ The expression levels of miRNAs in prion diseases have also been investigated in models such as the BSE-infected cynomolgus monkey, scrapie-infected mice and scrapie-infected cell lines.^8, 9, 10^ However, due to differences in experimental and analytic methodologies, the results seem to be in conflict.

Scrapie, the earliest recognized prion disease in the middle of the 18th century, is widely distributed worldwide with different morbidity in different regions. Scrapie has been adapted in various species of experimental rodents since the early 1960s. Scrapie-infected rodent models mimic the pathogenesis of natural prion infection to a great degree and function as valuable, indispensable and economical tools in the research field of prion diseases. Up until now, decades of research on scrapie-infected rodent models have been described with different incubation times and pathological features.^11^ To explore potential changes in brain miRNAs during prion infection, the expression profiles of miRNAs in the brains of three scrapie-infected rodent models, mice infected with strains 139A, ME7, and S15, were compared using second-generation deep sequencing technology. The similarities and differences in the differentially expressing miRNAs, the involved biological pathways and the novel miRNAs in control mice and those infected with the various scrapie models were analysed.

## MATERIALS AND METHODS

### Sample collection

All procedures involving live animals were approved by the Research Ethics Committee of the National Institute for Viral Disease Control and Prevention, China CDC.

Four C57BL mice infected with scrapie strains 139A or ME7, as well as four secondary passage mice that were infected with the lysates of SMB-S15 cells (S15)^12^ were used in this study. The procedure for the intracerebral inoculation of the scrapie agents into mice was described previously.^12, 13^ Briefly, under halothane anaesthesia, 28-day-old C57BL mice received an intracerebral inoculation with 1 μL of 10% brain homogenates from mice infected with the individual scrapie agents. The animals were monitored twice a week before clinical signs of infection were observed by experienced staff and then once per day after the appearance of the clinical symptoms until the animals died. At the end of the clinical phase, the animals were euthanized by ether and exsanguinated. Mice with intracerebral saline injections were used as normal controls and were sacrificed approximately 180 days post-inoculation. All brains were taken surgically; the cortical region of every infected or normal mouse was carefully separated and immediately placed in liquid nitrogen to freeze quickly. The incubation times of the mice infected with agents 139A, ME7 and S15 were 183.9±23.1, 184.2±11.8 and 153.0±2.2 days, respectively.^12^ The presence of proteinase K (PK)-resistant PrP^Sc^ in the brains of the infected mice was evaluated by PrP-specific Western blots; these results are shown in Supplementary Figure S1.

### Total RNA isolation

The total RNA of cortical tissue from each mouse was extracted with the commercial RNeasy mini kit (Qiagen, Hiden, Germany). The same amount of RNA from four mice was pooled for each group, including the 139A, ME7, S15 and control groups. The integrity of the RNA samples was determined by capillary electrophoresis on a Bioanalyzer (Agilent 2100, PaloAlto, CA, USA) according to the supplier's instructions. The RNA concentrations were determined photometrically at 260 nm on a NanoDrop (PeqLab, Erlangen, Germany), and 5 μL (5 μg) of each sample were applied to ultra-deep sequencing.

### Small RNA deep sequencing and bioinformatics analyses

Construction of cDNA libraries, cluster generation and subsequent ultra-deep sequencing on the Solexa/Illumina platform were performed at Beijing Genomics Institute Tech (BGI), Shenzhen, China. In brief, 5 μg of total RNA from each sample was size fractionated by 15% polyacrylamide gel electrophoresis. The small RNA fraction (18–30 nt) was extracted and ligated to 5′- and 3′-RNA adaptors using T4 RNA ligase. The RNA-adaptor constructs were purified and reverse transcribed. Then, the reverse transcribed products were amplified using the following PCR program: 98 °C for 30 s, followed by 15 cycles of 98 °C for 10 s, 72 °C for 15 s and 72 °C for 10 min. Ultra-deep sequencing was performed using one flow cell channel per sample.

The data analysis process routinely used in BGI is schematically summarized in Supplementary Figure S2. Briefly, the sequence tags from the ultra-deep sequencing first underwent the data cleaning analysis to remove low-quality tags and 5′-adaptor contaminants. The small clean RNA tags were mapped to the mouse genome using Short Oligonucleotide Analysis Package (SOAP) to analyse their expression and distribution in the genome.^14^ Meanwhile, the length distribution of the clean tags and common and specific sequences between samples were summarized. Using the Rfam 10.1 and GenBank database, the fragments of rRNA, scRNA, snoRNA, snRNA and tRNA were removed. Repeat-associated sRNAs and degradation fragments of mRNAs were also eliminated after small RNA tags were aligned to exons and introns of mRNA. The identified known miRNAs were subjected to the analyses for differential expression, cluster and the KEGG pathway. On the other hand, the reads that did not match the above databases were considered to be unannotated sRNAs and subjected to further processing as potential miRNAs with seed edit and novel miRNA prediction using the novel miRNA prediction software, MIREAP (http://sourceforge.net/projects/mireap/).

For the identification of the regulated miRNAs, the libraries derived from the individual scrapie-infected and control mice were normalized. Usually, two alternative methods were used for normalization. One method was the comparison of total small RNAs sequenced for each library. The other was the comparison of the total number of miRNAs in the respective samples. To avoid the bias created by the high proportion of miRNAs when normalized to the total number of miRNAs, we chose to normalize to the total count of the clean reads. The normalization formula was normalized expression=actual miRNA count/total count of clean reads and multiplied by 10^5^. Then, the changing fold, *P-*value (calculated by Poisson distribution) and *Q-*value of the normalized data were calculated. If one miRNA had no read, the normalized read count of this miRNA was set at 0.01. The miRNAs with more than a twofold change (showing in log2.0) and a Q value <0.01 compared with a normal control were considered differentially expressing miRNAs.

### Prediction of novel miRNAs

To identify genuine miRNAs from deeply sequenced small RNA libraries, a computational tool, MIREAP, was used in this study.^15, 16^ Some key points were considered during the analysis: (i) The tags which could be used to predict novel miRNAs were from unannotated tags that matched the reference genome, tags that could be aligned to an intron region and the tags that could be aligned to an antisense exon region. (ii) Those genes whose sequences and structures satisfied the two criteria, hairpin miRNAs that can fold secondary structures and mature miRNAs that are present in one arm of the hairpin precursors, were considered to be candidate miRNA genes. (iii) The mature miRNA strand and its complementary strand (miRNA*) contained 2-nucleotide 3′ overhangs. (iv) Hairpin precursors lacked large internal loops or bulges. (v) The secondary structures of the hairpins were steady, with the free energy of hybridization lower than or equal to −18 kcal/mol. (vi) There must have been no fewer than five mature miRNAs with predicted hairpin structures in the alignment result. The comparison of the expression of novel miRNAs in the infected animal and mock animals was conducted with the same protocol used for the known miRNAs described above. All remaining candidates were searched against the miRBase 20.0 to rule out known miRNAs.

### MiRNA verification by quantitative real-time PCR

Real-time quantitative PCR with reverse transcription (qRT-PCR) assays were performed using the All-in-One miRNA qRT-PCR Reagent Kits (GeneCopoeia Company, Rockville, MD, USA). The specific primers for mmu-miR-146a-5p, mmu-miR-341-3p, mmu-miR-879-5p, mmu-miR-3470a, mmu-miR-3473a, mmu-miR-3473b, mmu-miR-96-5p, mmu-miR-141-3p, mmu-miR-182-5p, mmu-miR-200a-3p, mmu-miR-200b-3p, mmu-miR-200b-5p, mmu-miR-2 and mmu-miR-20 were synthesized by the GeneCopoeia Company, Beijing, China. The mixture of the PCR reaction contained 2 μL of first-strand cDNA, 10 μL of the 2 × all-in-one qmix, 2 μL of the all-in-one primer, 2 μL of the universal adapter primer, 0.4 μL of 50 × ROX Dye and 3.6 μL ddH_2_O in a final volume of 20 μL. After initial denaturation at 95 °C for 10 min, cycling was performed as follows: 95 °C for 10 s and 60 °C for 45 s for 40 cycles in a 7500 Real-Time PCR System (Applied Biosystems, PoloAlto, CA, USA). The qRT-PCR for each miRNA was repeated more than three times.

## RESULTS

The extracted total RNA, containing miRNA, from mice infected by three different scrapie strains as well as normal mice was subjected to Solexa sequencing after passing the RNA quality control. Clean reads (excluding the reads containing ambiguous base and adaptor contaminants) yielded by the Solexa sequencing were analysed further. A total of 11 782 491 clean reads (98.89%) in the 139A group, 11 673 078 (97.89%) in the ME7 group, 11 164 603 (93.94%) in theS15 group and 11 849 236 (99.28%) in the control group were obtained (Supplementary Table S1). The length distributions of the majority of the reads in all tested groups were in the range of 18–24 nt, with the peak at 22 nt long. Assays of the common and unique tags and total sRNAs shared between each scrapie-infected and control mouse showed that the percentages of the unique sRNAs shared between the control and 139A, ME7 or S15 groups were 8.37%, 9.29% and 6.36%, respectively, while those of the common total sRNAs shared between the control and 139A, ME7 or S15 groups were 91.09%, 92.02% and 87.22%, respectively, highlighting an overall consistency in the sequencing among the samples tested.

To annotate the small RNA tags, the sequencing data were analysed by SOAP. Approximately, 72.02%, 73.32%, 67.57% and 74.32% of the total reads were matched to the genome in the 139A, ME7, S15 and control groups, respectively. After alignment to the GenBank database from Rfam (http://rfam.xfam.org/), all sRNAs were classified into different categories (Supplementary Table S2). In total, 823, 871, 799 and 804 known miRNAs were qualified in the 139A, ME7, S15 and normal control groups, respectively.

### The miRNAs up- and downregulated in the mouse brains infected with three scrapie strains

**Figure 1:**
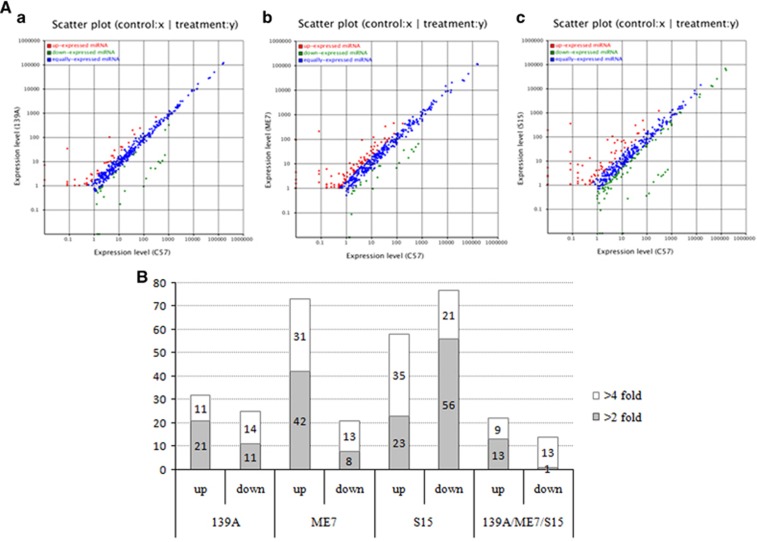
(**A**) Scatter plots of the differential expression levels of the miRNAs between the 139A- (a), ME7- (b), S15- (c) infected mice and mock-infected mouse. Red spots show the upregulated miRNAs, green ones show the downregulated miRNAs and blue ones show the miRNAs whose expression levels were unchanged. The expression levels (log2) of the infected samples (139A, ME7 and S15) are indicated in the Y axis, while that of the normal control is in the *X* axis. (**B**) Distributions of the upregulated and downregulated differentially expressed miRNAs in the 139A, ME7 and S15 groups. Grey columns represent the number of miRNAs with at least a fourfold change, while white ones represent those with at least a twofold changed. The *Y* axis represents the number of miRNA changes in different samples. The number of changed miRNAs in the individual groups is indicated inside the columns.

Using the strategy described above, scatter plots of the differential expressions of the miRNAs between the individual infected mice and control mice were generated separately. As shown in Figure 1A, the majority of the identified miRNAs maintained similar expression levels (blue dots) between the infected and control mice. Further analysis revealed that the number of miRNAs significantly up- and downregulated in the 139A, ME7 and S15 groups were 32 (11 higher than fourfold) and 25 (14 lower than fourfold), 73 (31 higher than fourfold) and 21 (13 lower than fourfold), 58 (35 higher than fourfold) and 77 (21 lower than fourfold), respectively (Figure 1B). The most significantly up- and downregulated miRNAs in scrapie-infected mice were mmu-miR-3473e (9.48 log2) and mmu-miR-141-5p (−7.33 log2) in the 139A group, mmu-miR-3473e (13.18 log2) and mmu-miR-200a-5p (−7.08 log2) in the ME7 group, and mmu-miR-3473e (14.15 log2) and mmu-miR-183-3p (−10.15 log2) in the S15 group. The changed miRNAs in each infected group are given in Table 1, with the changed folds (log2) and Q values. The reads numbers of the known miRNAs in the brains of the four groups are listed in Supplementary Table 3.

The miRNAs commonly changed in the three types of scrapie-infected mice were selected. As shown in Figure 1B and Table 1, 22 miRNAs were commonly upregulated and 14 miRNAs were commonly downregulated. Among them, nine miRNAs showed more than a fourfold upregulation, including mmu-miR-341-3p, mmu-miR-3473a, mmu-miR-3473b, mmu-miR-3473e, mmu-miR-5100, mmu-miR-5121, mmu-miR-690, mmu-miR-709 and mmu-miR-879-5p, while 13 miRNAs revealed more than a fourfold downregulation, including mmu-miR-141-3p, mmu-miR-141-5p, mmu-miR-182-3p, mmu-miR-182-5p, mmu-miR-183-3p, mmu-miR-183-5p, mmu-miR-200a-3p, mmu-miR-200a-5p, mmu-miR-200b-3p, mmu-miR-200b-5p, mmu-miR-200c-3p, mmu-miR-429-3p and mmu-miR-96-5p.

### Identification of novel miRNAs in the mouse brains infected with three scrapie strains

The sequences that did not match any database were considered to be unannotated sequences. There were 2 024 076 (17.18%) unannotated reads of small RNAs in the 139A group, 1 974 980 (16.92%) in the ME7 group, 1 529 429 (13.7%) in the S15 group and 2 289 457 (19.32%) in the healthy control. Using the novel miRNA prediction software, MIREAP, we obtained 15 novel miRNA precursor candidates in 139A-infected mice, 20 in ME7-infected ones, 21 in S15-infected ones and 16 in the normal control group. Compared with the data from the normal control, three novel miRNAs were significantly downregulated in the 139A group, two up- and two downregulated in the ME7 group, and two up- and three downregulated in the S15 group (Table 2). Among them, two novel miRNAs, novel-mir-2 and novel-mir-20 were commonly found to be downregulated in all three infected groups. The downregulated log2-folds of novel-mir-2 in the 139A, ME7 and S15 groups were 2.72, 2.68 and 4.06, while those of novel-mir-20 were 6.84, 6.78 and 6.28, respectively. In addition, novel-mir-17 was found to be commonly downregulated in the 139A and S15 groups, but not in the ME7 groups. The reads numbers of the novel miRNAs in four groups are listed in Supplementary Table S4.

### Validation of the known 12-miRNA and novel 2-miRNA signature by qRT-PCR

To validate the up- and downregulated miRNAs identified in the Solexa sequencing, 6 upregulated (mmu-miR-146a-5p, mmu-miR-341-3p, mmu-miR-879-5p, mmu-miR-3470a, mmu-miR-3473a and mmu-miR-3473b) and six downregulated miRNAs (mmu-miR-96-5p, mmu-miR-141-3p, mmu-miR-182-5p, mmu-miR-200a-3p, mmu-miR-200b-3p and mmu-miR-200b-5p), as well as two novel miRNAs (novel-mir-2 and novel-mir-20), were selected. The transcriptional levels of those miRNAs in the brains of 139A-, ME7- and S15-infected mice were separately evaluated with individual specific qRT-PCRs, using snRNA U6 as the internal control. The mean value of each special miRNA in each animal group was from the averaged data of four tested animals after normalization with that of the internal control. The relative expression of each special miRNA in each infectious animal model was calculated with those of the control mice.

**Figure 2:**
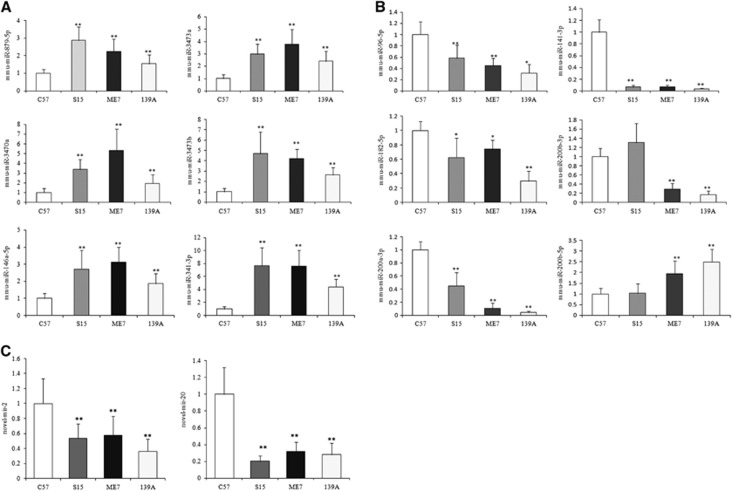
Analysis of the expression levels of the selected miRNAs in the brains of 139A-, ME7- and S15-infected mice compared with that of the normal control mice by individual qRT-PCR. The *Y* axis represents the fold change of the miRNAs. (**A**) Six upregulated miRNAs. (**B**) Six downregulated miRNAs. (**C**) Two downregulated novel miRNAs. The data for each miRNA in each tested group are the average of four individual mice. Each test was repeated three times. Data are presented as the mean and s.d. *P<0.05, **P<0.01.

For the upregulated miRNAs found in the deep sequencing analysis, the expression levels of all six miRNAs selected were increased in the brains of the three infectious mouse models, and the qRT-PCR analysis revealed significant differences (Figure 2A). For the six downregulated miRNAs selected from the Solexa sequencing, four of them showed significant reductions in the expression levels in the brains of the three infectious models, while one (mmu-miR-200b-3p) was downregulated in the 139A- and ME7-infected mice but upregulated in the S15-infected group, and another (mmu-miR-200b-5p) was upregulated in 139A- and ME7-infected mice and almost unchanged in the S15-infected group (Figure 2B). In addition, two novel miRNAs whose expression levels were downregulated in the scrapie-infected animals with the Solexa sequencing showed significant reductions in expression level in qRT-PCR as well (Figure 2C). These data highlight a good correlation between the miRNA expression profile detected by deep sequencing and qRT-PCR.

### Similarity in the global expression of miRNAs in the mouse brains infected with three scrapie strains

**Figure 3:**
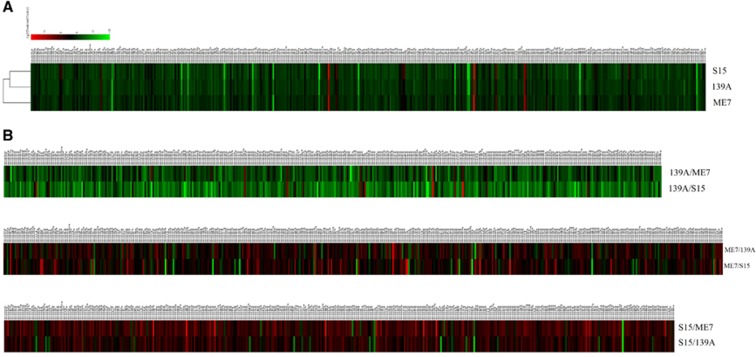
Hierarchical clustering analysis of the differentially expressed miRNAs. (**A**) 139A-, ME7- and S15-infected mice vs normal control. (**B**) ME7 and S15 vs 139A; 139A and S15 vs ME7; ME7 and 139A vs S15.

To see the similarity and diversity among the mice infected with different scrapie strains, all identified known miRNAs were compared in a pairwise method. Scatter plot assays show that the expression levels of most of the miRNAs among the mice infected with the three agents were similar (blue dots, Figure 1A). To explore the relationship of the changed known miRNAs among the brain tissue infected with the three scrapie strains, all identified miRNAs were subjected to a hierarchical clustering analysis after being normalized to the value of each miRNA in the normal control group. This analysis revealed a great similarity among the miRNA expression profiles of the mice infected with the three different scrapie strains, in which the profile of the 139A-infected mice seemed closer to that of the S15-infected mice than to the ME7-infected mice (Figure 3A). Furthermore, the miRNA expression profiles of the three scrapie infections were analysed with pairwise comparisons. As shown in Figure 3B, the expression levels of the tested miRNAs in the 139A group were generally higher than those in the ME7 and S15 groups. These data indicate that the miRNA expression tendencies in the brains of mice infected with three different scrapie strains are overall the same but still possess some strain specificities.

### Potential involvement of significant pathways in the mouse brains infected with three scrapie strains

To consider the possible affected biological pathways of the differentially expressed miRNAs, the Kyoto Encyclopedia of Genes and Genomes (KEGG) pathway analysis was conducted. More than 300 biological pathways were involved but only 15, 13 and 13 pathways were shown to be significantly affected (*P*<0.05; Table 3). Twelve of them were commonly observed in all three groups, including olfactory transduction, metabolic pathways, bacterial invasion of epithelial cells, *Staphylococcus aureus* infection, the cytosolic DNA-sensing pathway, Jak–STAT signalling pathway, inositol phosphate metabolism, complement and coagulation cascades, measles, primary immunodeficiency, epithelial cell signalling in *Helicobacter pylori* infection and intestinal immune network for IgA production, indicating a great similarity in the infections with the different scrapie agents. High coincidence of the potentially affected pathways in the brains from mice infected with three different scrapie strains reflects the similar pathogenic processes.

## DISCUSSION

The development of new-generation deep sequencing technology has greatly expanded the understanding of miRNA in many areas. Obtaining large quantities of data provides the possibility to explore and reveal the overall miRNA expression profiles of entire organisms and tissues. Comprehensive analysis of miRNA expression between prion-infected and healthy brain tissue or among the brains infected with various prion strains will help us to better understand the role of miRNAs in the development of the disease as well as to find novel biomarkers for diagnosis and therapy. In the current study, we show the miRNA expression profiles in the brains of three mouse models infected with different scrapie strains. We have found 22 up- and 14 downregulated known miRNAs common to all three animal models.

Our hierarchical clustering analysis identifies similarities in the brain miRNA expression profiles of the three different groups of scrapie-infected mice compared to that of normal control mice. Moreover, the KEGG pathway analysis further illustrates great commonalities in the biological pathways in which the altered miRNAs are involved. This analysis reflects not only a similar neuropathological abnormality among the infections of various prion strains but also the high credibility of the technique. Scrapie agent 139A- and ME7-infected mice are widely used rodent models, which show great similarities in clinical, pathological and pathogenic characteristics.^17^ However, they exhibit different incubation times in the interspecies infection from mouse to hamster, where ME7 shows a longer incubation time, although their incubation periods become quite comparable with subsequent passages.^18^ The brain proteomic assays from our group have recently identified a larger number of differentially expressed proteins in ME7-infected mice (Shi *et al.* unpublished data). Coincidental with the observations from the proteomics analyses, ME7-infected mice contain more significantly changed brain miRNAs than 139A-infected mice. The scrapie strain S15-infected mouse model was established by us via intracerebral inoculation of the prion agents from SMB-S15 cells into C57BL mice. Compared with ME7- and 139A-infected mice, S15-infected mice display relatively short incubation times. More significantly changed miRNAs are observed in the brains of S15 mice.

Previous studies have proposed an increase in miRNA-146a in human brain cells that is believed to be in response to pathophysiological and oxidative stress in viral infection and some nervous system disorders.^19, 20^ In our results, brain miRNA-146a is also upregulated in all three models of scrapie-infected mice, with an average fold-change value of 2.57. Numerous data show that human miRNA-146a, which is involved in stress and neuropathology, is mediated by NF-кB.^21, 22, 23^ Three canonical tandem NF-кB binding sites in the pre-miRNA-146a promoter suggest that miRNA-146a might be the miRNA most responsive to NF-кB. In AD patients, miRNA-146a is greatly upregulated compared with all other NF-кB-regulated miRNAs; meanwhile, the NF-кB level is also significantly increased.^24^ Our preliminary data also illustrate an increase in NF-кB in scrapie 139A-infected mouse brains (data not shown). In addition, studies based on the brain tissue of AD patients have shown an upregulation of miRNA-146a, which targets and downregulates complement factor H (CFH), interleukin-1 receptor-associated kinase-1 (IRAK-1) and tetraspanin-12 (TSPAN12). CFH and IRAK-1 are believed to be related to neuroinflammation of the nervous system, while TSPAN12 is associated with amyloidogenesis.^25, 26^ Activation of the microglia and complement system and increases in inflammatory cytokines have been repeatedly observed in human and animal brains with prion diseases.^27^ More work need to be conducted to clarify the relationship of miR-146a with neuroinflammation in TSEs.

Mmu-miR-3473b was shown to be remarkably increased in all three scrapie-infected mouse models, with an average fold-change value of 10.6. A recent study has shown that miR-3473b is responsible for downregulation of IFN-γ-priming macrophage activation.^28^ In this case, increasing miR-3473b downregulates its target protein, phosphatase and tensinhomologue, which then inhibits the PI3K/Akt/IL-10-negative loop and finally promotes an inflammatory response and classical activation of macrophages. Activation of microglia (macrophages in the nervous system) has been repeatedly observed in the brain tissue of prion-infected experimental rodents and sporadic CJD patients.^27^ The relationship between increased miR-3473b expression and activation of microglia during prion infection deserves further study.

In addition to mmu-miR-3473b, other two family numbers, mmu-miR-3473a and mmu-miR-3473e, are also greatly increased in the brains of the three scrapie-infected mouse models, with average fold-change values of 5.3 and 12.3, respectively. A search for the possible targets of mmu-miR-3473a in MIRDB (http://www.mirdb.org) identified flotillin 2 (Flot-2) as one of the target genes with the highest score. Flotillins are membrane association proteins, consisting of two homologous members, flotillin-1 and flotillin-2. Our previous study illustrates that flotilin-1 can mediate PrP^C^ endocytosis in the cultured cells during Cu^2+^ stimulation through a molecular interaction.^29^ Further understanding of the contribution of the high brain level of mmu-miR-3473a during prion infection to flotillin-mediated PrP^C^ endocytosis will help us further our knowledge of the physical biology and pathology of prion disease.

We have also found that five members of the miR-200 family are markedly decreased in the brains of scrapie-infected mice, including mmu-miR-200a-3p, mmu-miR-200a-5p, mmu-miR-200b-3p, mmu-miR-200b-5p and mmu-miR-200c-3p. Recent studies have shown that the miR-200 family is also significantly reduced during clinical disease in the scrapie agent RML (RML, mouse prion strain from Rocky Mountain Laboratory)-infected mouse neural synapses.^30^ The miR-200 family is a cluster of miRNAs closely linked to the epithelial–mesenchymal transition (EMT) and is believed to have an essential role in tumour suppression by inhibiting EMT, the initiating step of metastasis.^31^ A number of experimental studies show that changes in miR-200 family levels have been associated with enhanced tumorigenesis and are significantly correlated with decreased survival caused by lung cancer and other cancers.^32^ Many studies have reported that PrP^C^ can stimulate the outgrowth of neurites. Although the exact function of PrP^C^ is unknown, several lines of evidence suggest that PrP^C^ is involved in cell adhesion, migration, proliferation, differentiation, ion homeostasis and signal transduction. All of these functions imply that PrP may be involved in tumorigenesis.^31^ The role of the miR-200 family in TSEs is not very clear.

We have also identified several novel miRNAs in this study. Specifically, two novel miRNAs (novel-mmu-miR-2 and novel-mmu-miR-20) are commonly changed in all three animal models. qRT-PCR validation shows that expression of both novel miRNAs is reduced in the brains of the three scrapie-infected mouse models, which is consistent with the results of the Solexa sequencing. Predicting the target proteins of these two novel miRNAs will be helpful for understanding their role in prion biology.

Increasing amounts of data suggest the important role of miRNA in TSEs. For example, combined with mRNA expression profiles, ultra-deep sequencing analysis has found the changes of miRNA in N2a cells is related with cholesterol metabolism.^10^ MiRNA expression profiles in exosomes of prion-infected neuronal cells also show a distinct signature compared to the control cells.^33^ Using a scrapie-infected mouse model, the expression difference of miRNAs in synapses has also been studied along with disease progression.^30^ Although there are often marked differences in the results among different experiments due to different methodological techniques and biological specimens, there are still many similarities in the miRNA expression profiles. Comprehensive analysis of these miRNA profiles will not only provide clues for the study of the mechanisms of TSEs but will also be beneficial in the search for possible diagnostic marker(s) in the future.

## Figures and Tables

**Table 1:** Fold changes (log2 Diseased/Ctrl) of commonly changed miRNAs in three scrapie infected mice compared with normal mice

	**139A**		**ME7**		**S15**		**Average**
	**Fold change**	***Q*-values**	**Fold change**	***Q*-values**	**Fold change**	***Q*-values**	**Fold change**
*Upregulated*							
mmu-miR-1291	1.73069501	0.000961380	1.86965881	0.00019267	1.51890893	0.004970014	1.706421
mmu-miR-142-5p	1.29265354	2.26E−40	1.63458319	1.09E−71	1.29247727	1.26E−39	1.406571
mmu-miR-146a-5p	1.78876097	2.24E−44	3.32695498	1.35E−260	2.59501539	2.63E−120	2.570244
mmu-miR-1940	1.71215902	1.64E−09	1.84877485	1.92E−11	4.87650052	6.64E−221	2.812478
mmu-miR-217-5p	1.56453958	0.000117246	1.36040648	0.00126549	2.12767033	2.79E−09	1.684205
mmu-miR-27a-3p	1.55040502	2.59E−35	2.44451335	2.06E−117	2.07908094	7.51E−74	2.024666437
mmu-miR-298-5p	1.64310969	1.46E−125	1.10279037	2.36E−47	1.71058683	2.01E−136	1.48549563
mmu-miR-3068-3p	1.54346801	1.34E−15	1.25292599	1.03E−09	2.1867832	3.69E−37	1.661059067
mmu-miR-331-3p	1.21709726	3.98E−19	1.94958185	8.10E−61	1.47013113	2.14E−29	1.545603413
mmu-miR-341-3p	2.47575721	4.70E−316	3.21977269	0	3.09062477	0	2.928718223
mmu-miR-3470a	2.10950917	4.21E−39	1.94182946	2.36E−31	3.46331943	1.71E−165	2.50488602
mmu-miR-3470b	1.74983844	7.77E−19	1.85242729	1.11E−21	3.47027695	7.16E−129	2.357514227
mmu-miR-3473a	3.81539088	0.001292487	5.90415303	1.62E−16	6.51201489	1.67E−25	5.4105196
mmu-miR-3473b	8.64828516	6.81E−118	11.25691574	0	12.05225858	0	10.65248649
mmu-miR-3473e	9.47759638	2.41E−25	13.17555891	0	14.19021127	0	12.28112219
mmu-miR-381-5p	1.47444394	4.05E−08	1.97333721	3.10E−16	1.43783296	1.09E−07	1.628538037
mmu-miR-5100	7.40718277	3.11E−06	8.82667559	3.34E−16	7.80683958	3.62E−08	8.01356598
mmu-miR-5121	3.59305917	0.004163558	4.34339098	2.46E−05	5.67071497	6.74E−14	4.535721707
mmu-miR-540-5p	1.62285265	9.01E−05	2.94046567	5.01E−21	2.62501921	3.93E−15	2.39611251
mmu-miR-690	4.61743631	3.65E−287	4.48637937	6.24E−257	4.65849988	4.05E−292	4.58743852
mmu-miR-709	2.59305917	0.014963063	2.60645346	0.01222732	4.25568866	2.79E−09	3.151733763
mmu-miR-879-5p	3.40035339	9.98E−05	5.17125389	8.59E−19	5.40768057	2.43E−22	4.659762617

*Downregulated*							
mmu-miR-141-3p	−4.86398081	8.70E−51	−2.65784566	5.78E−31	−6.00869028	8.25E−54	−4.51017225
mmu-miR-141-5p	−7.32508054	6.62E−06	−4.22578534	6.15E−05	−7.32508054	8.13E−06	−6.29198214
mmu-miR-182-3p	−2.89910497	0.003267799	−6.98401964	8.64E−05	−6.98401964	0.000105067	−5.622381417
mmu-miR-182-5p	−5.75925828	0	−3.89074488	0	−7.49598503	0	−5.715329397
mmu-miR-183-3p	−6.06902997	5.90E−37	−3.5958301	2.16E−27	−10.15394469	6.23E−39	−6.606268253
mmu-miR-183-5p	−5.82811208	0	−4.04911813	0	−6.79930575	0	−5.55884532
mmu-miR-200a-3p	−5.29461539	0	−3.47189439	0	−7.38682949	0	−5.384446423
mmu-miR-200a-5p	−7.07713616	4.85E−05	−7.07713616	4.43E−05	−3.91363743	0.000468688	−6.022636583
mmu-miR-200b-3p	−5.31967659	0	−3.36329472	0	−7.39949571	0	−5.36082234
mmu-miR-200b-5p	−6.71479583	0	−3.89399342	5.58E−245	−8.09643234	0	−6.235073863
mmu-miR-200c-3p	−5.16308038	0	−2.41730379	1.43E−294	−7.1326953	0	−4.904359823
mmu-miR-429-3p	−5.72602717	0	−3.58655409	0	−7.37766838	0	−5.563416547
mmu-miR-486-3p	−1.50083416	4.30E−06	−1.66430936	5.82E−07	−1.80163716	1.52E−07	−1.65559356
mmu-miR-96-5p	−5.98992488	0	−3.53420691	2.90E−286	−8.11395151	0	−5.8793611

**Table 2:** Identification of new miRNAs in the scrapie-infected mice brains

**Sample**	**No. of potential new miRNA candidates**	**Differential expressed new miRNA**				
		**No.**	**Name of miRNA**	**Fold change (log2 Infected/Ctrl)**	***Q*-values**	**Sequence**
Ctrl	16	—	—	—	—	—
139A	15	3	novel-mir-2	−2.72316688	1.62E−65	TCT GGA CAC ATG TGG CTT TT^a^
			novel-mir-17	−10.0883642	5.30E−39	TTG GGA AGG TGG ATA ATT TGG^b^
			novel-mir-20	−6.84447582	0.000106	AGC TGC GGT AGG AAG GAT GCG G^c^
ME7	20	4	novel-mir-2	−2.68733439	5.21E−64	
			novel-mir-20	−6.78447582	0.000137	
			novel-mir-28	7.68369645	1.32E−07	AGG GGG TGG GGG GTT TGG AG
			novel-mir-36	7.68369645	1.32E−07	TCG TGA CTG TAC TTG GTA TT
S15	21	5	novel-mir-2	−4.0604619	2.70E−90	
			novel-mir-17	−10.0883642	2.32E−37	
			novel-mir-20	−6.28447582	0.000133	
			novel-mir-38	9.20738044	3.14E−21	TTG GGA AGG TGG ATA ATT TG
			novel-mir-49	9.31274685	1.12E−22	AAT GCT AGA CAA AGT GCG GGG G

a novel-mir-2: commonly changed in all three infected samples.

b novel-mir-17: commonly changed in 139A and S15.

c novel-mir-20: commonly changed in all three infected samples.

**Table 3:** The potential involved KEEG pathways

	**No. of the involved pathways (*P*<0.05)**	**Pathway**	**Target genes with pathway annotation (22 115)**	**All genes of the species with pathway annotation (22 876)**	***P-*values**	***Q-*values**	**Pathway ID**
1139A	15	Olfactory transduction	1248 (5.64%)	1256 (5.49%)	5.617129e−11	1.735693e−08	ko04740
		Metabolic pathways	2148 (9.71%)	2202 (9.63%)	0.007657089	6.489340e−01	ko01100
		Bacterial invasion of epithelial cells	251 (1.13%)	253 (1.11%)	0.0088366	6.489340e−01	ko05100
		*Staphylococcus aureus* infection	133 (0.6%)	133 (0.58%)	0.01096616	6.489340e−01	ko05150
		Cytosolic DNA-sensing pathway	131 (0.59%)	131 (0.57%)	0.01173853	6.489340e−01	ko04623
		Jak–STAT signalling pathway	325 (1.47%)	329 (1.44%)	0.01398002	6.489340e−01	ko04630
		Inositol phosphate metabolism	123 (0.56%)	123 (0.54%)	0.01541075	6.489340e−01	ko00562
		Complement and coagulation cascades	227 (1.03%)	229 (1%)	0.01680088	6.489340e−01	ko04610
		Measles	288 (1.3%)	292 (1.28%)	0.03225006	9.315842e−01	ko05162
		Primary immunodeficiency	92 (0.42%)	92 (0.4%)	0.04420676	9.315842e−01	ko05340
		Epithelial cell signalling in *Helicobacter pylori* infection	143 (0.65%)	144 (0.63%)	0.04515162	9.315842e−01	ko05120
		Intestinal immune network for IgA production	91 (0.41%)	91 (0.4%)	0.04573424	9.315842e−01	ko04672
		Valine, leucine and isoleucine degradation	90 (0.41%)	90 (0.39%)	0.04731444	9.315842e−01	ko00280
		Hepatitis C	308 (1.39%)	313 (1.37%)	0.04914583	9.315842e−01	ko05160
		Toll-like receptor signalling pathway	227 (1.03%)	230 (1.01%)	0.04997359	9.315842e−01	ko04620
ME7	13	Olfactory transduction	1248 (5.64%)	1256 (5.49%)	1.263096e−10	3.902967e−08	ko04740
		Bacterial invasion of epithelial cells	251 (1.13%)	253 (1.11%)	0.01030823	7.434235e−01	ko05100
		*Staphylococcus aureus* infection	133 (0.6%)	133 (0.58%)	0.01214988	7.434235e−01	ko05150
		Metabolic pathways	2148 (9.71%)	2202 (9.63%)	0.01289161	7.434235e−01	ko01100
		Cytosolic DNA-sensing pathway	131 (0.59%)	131 (0.57%)	0.01298553	7.434235e−01	ko04623
		Jak–STAT signalling pathway	325 (1.47%)	329 (1.44%)	0.0165815	7.434235e−01	ko04630
		Inositol phosphate metabolism	123 (0.56%)	123 (0.54%)	0.01694278	7.434235e−01	ko00562
		Complement and coagulation cascades	227 (1.03%)	229 (1%)	0.01924721	7.434235e−01	ko04610
		Regulation of actin cytoskeleton	711 (3.21%)	726 (3.17%)	0.03592082	9.695122e−01	ko04810
		Measles	288 (1.3%)	292 (1.28%)	0.03721578	9.695122e−01	ko05162
		Primary immunodeficiency	92 (0.42%)	92 (0.4%)	0.04745194	9.695122e−01	ko05340
		Intestinal immune network for IgA production	91 (0.41%)	91 (0.4%)	0.0490537	9.695122e−01	ko04672
		Epithelial cell signalling in *Helicobacter pylori* infection	143 (0.65%)	144 (0.63%)	0.04948214	9.695122e−01	ko05120
S15	13	Olfactory transduction	1248 (5.64%)	1256 (5.49%)	1.263096e−10	3.902967e−08	ko04740
		Bacterial invasion of epithelial cells	251 (1.13%)	253 (1.11%)	0.01030823	7.434235e−01	ko05100
		*Staphylococcus aureus* infection	133 (0.6%)	133 (0.58%)	0.01214988	7.434235e−01	ko05150
		Metabolic pathways	2148 (9.71%)	2202 (9.63%)	0.01289161	7.434235e−01	ko01100
		Cytosolic DNA-sensing pathway	131 (0.59%)	131 (0.57%)	0.01298553	7.434235e−01	ko04623
		Jak–STAT signalling pathway	325 (1.47%)	329 (1.44%)	0.0165815	7.434235e−01	ko04630
		Inositol phosphate metabolism	123 (0.56%)	123 (0.54%)	0.01694278	7.434235e−01	ko00562
		Complement and coagulation cascades	227 (1.03%)	229 (1%)	0.01924721	7.434235e−01	ko04610
		Regulation of actin cytoskeleton	711 (3.21%)	726 (3.17%)	0.03592082	9.695122e−01	ko04810
		Measles	288 (1.3%)	292 (1.28%)	0.03721578	9.695122e−01	ko05162
		Primary immunodeficiency	92 (0.42%)	92 (0.4%)	0.04745194	9.695122e−01	ko05340
		Intestinal immune network for IgA production	91 (0.41%)	91 (0.4%)	0.0490537	9.695122e−01	ko04672
		Epithelial cell signalling in *Helicobacter pylori* infection	143 (0.65%)	144 (0.63%)	0.04948214	9.695122e−01	ko05120
