# Supplementary material for: MiRNA expression profiles in the brains of mice infected with scrapie agents 139A, ME7 and S15
Source: Emerg Microbes Infect. 2016 Nov 9;5(11):e115–. doi: 10.1038/emi.2016.120 (PMC5148024; doi:10.1038/emi.2016.120)
Supplement: Supplementary Figure 1 [file emi2016120x1.pdf]

Supplemental Figure 1.

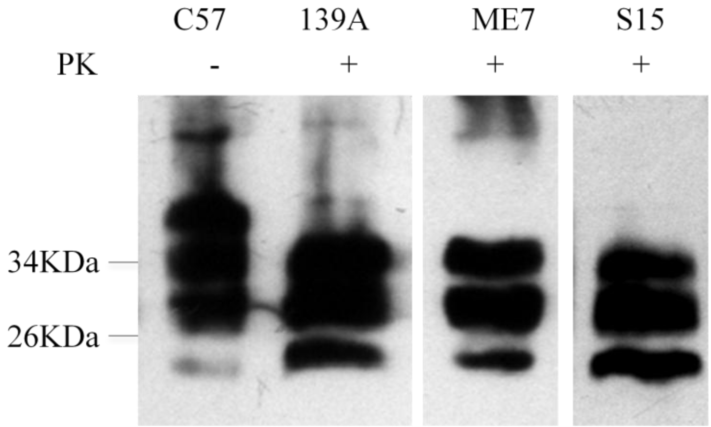

Supplementary Figure S1 Representative Western blots of PK-resistant PrP^{Sc} in the brains of the mice infected with scrapie agents 139A, ME7 or S15. Here, 10% various brain homogenates treated with 50 mg/mL PK (PK+) or without PK (PK-) were separated with 15% SDS-PAGE and blotted with PrP-specific monoclonal antibody 6H4[12]. The molecular markers are shown on the left.
