# Supplementary figures and images for: MiRNA expression profiles in the brains of mice infected with scrapie agents 139A, ME7 and S15
Source: Emerg Microbes Infect. 2016 Nov 9;5(11):e115–. doi: 10.1038/emi.2016.120 (PMC5148024; doi:10.1038/emi.2016.120)

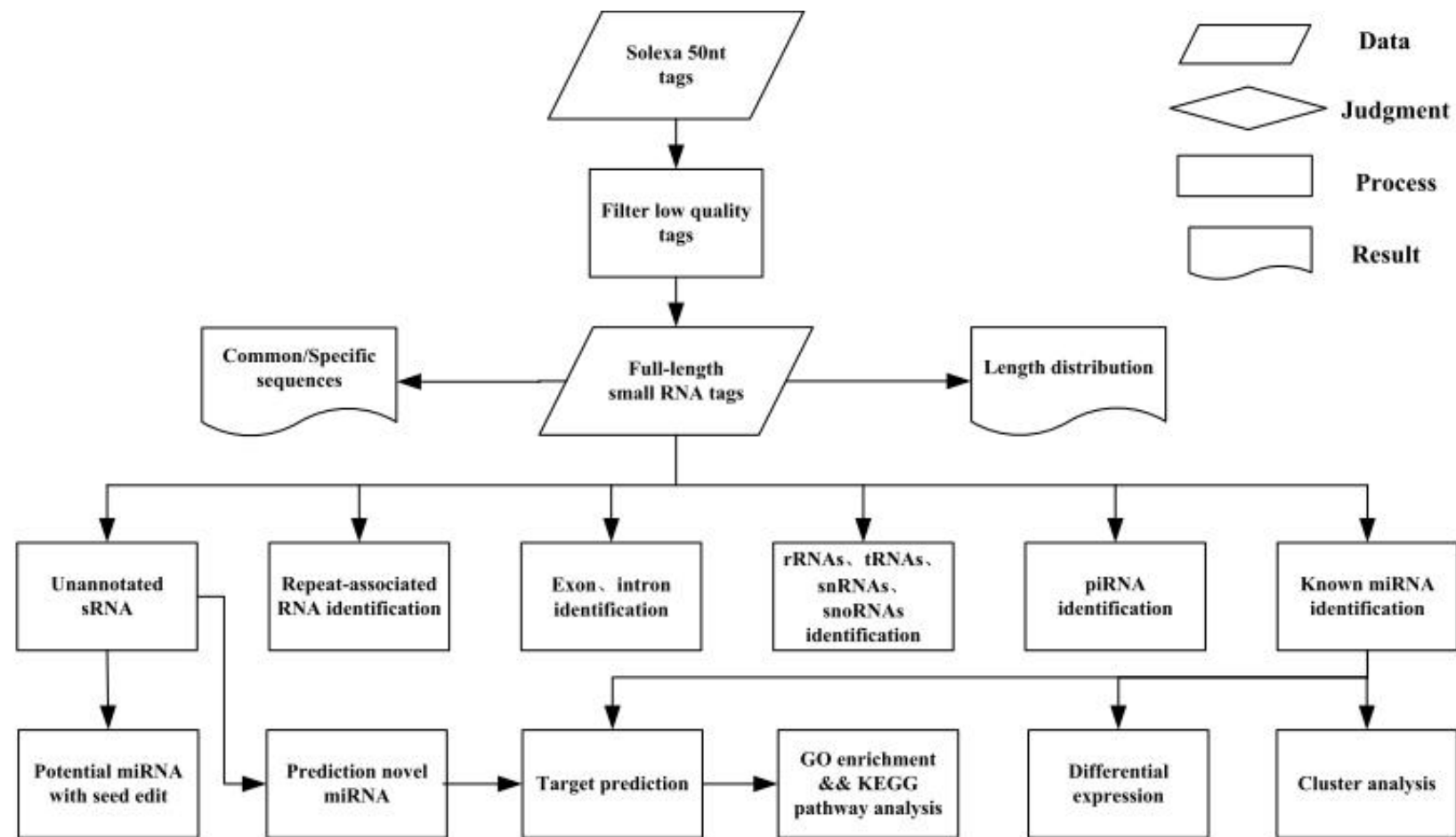

**Supplementary Figure S2** Data processing flow chart of miRNAs.

Supplement: Supplementary Figure 2 [file emi2016120x2.pdf]
