# Supplementary material for: MiRNA expression profiles in the brains of mice infected with scrapie agents 139A, ME7 and S15
Source: Emerg Microbes Infect. 2016 Nov 9;5(11):e115–. doi: 10.1038/emi.2016.120 (PMC5148024; doi:10.1038/emi.2016.120)
Supplement: Supplementary Table 1 [file emi2016120x3.pdf]

Supplementary Table S1 Summary of data cleaning

Items	Ctrl		139A		ME7		S15	
	numbers	0.00%	numbers	0.00%	numbers	0.00%	numbers	0.00%
total reads	12000000		12000000		12000000		12000000	
high quality	11934983	100	11914868	100%	11924422	100	11885292	100
3'adapter null	14099	0.12	19352	0.16	13928	0.12	18847	0.16
insert null	3482	0.03	3313	0.03	4973	0.04	5400	0.05
5'adapter contaminants	18231	0.15	24413	0.2	18808	0.16	15674	0.13
smaller than 18nt	49607	0.42	84869	0.71	213120	1.79	680322	5.72
polyA	328	0	430	0	515	0	446	0
clean read	11849236	99.28	11782491	98.89	11673078	97.89	11164603	93.94
