# Supplementary material for: MiRNA expression profiles in the brains of mice infected with scrapie agents 139A, ME7 and S15
Source: Emerg Microbes Infect. 2016 Nov 9;5(11):e115–. doi: 10.1038/emi.2016.120 (PMC5148024; doi:10.1038/emi.2016.120)
Supplement: Supplementary Table 2 [file emi2016120x4.pdf]

**Supplementary Table S2** Read abundances of small RNAs in three infected mice brain and health mouse libraries.

| Items | Ctrl | | 139A | | ME7 | | S15 | |
|---|---|---|---|---|---|---|---|---|
| | Total sRNAs | % | Total sRNAs | % | Total sRNAs | % | Total sRNAs | % |
| Total | 11849236 | 100% | 11782491 | 100% | 11673078 | 100% | 11164603 | 100% |
| exon_antisense | 1074 | 0.01% | 3612 | 0.03% | 3674 | 0.03% | 6797 | 0.06% |
| exon_sense | 71004 | 0.6% | 507844 | 4.31% | 384343 | 3.29% | 568502 | 5.09% |
| intron_antisense | 4681 | 0.04% | 33982 | 0.29% | 32803 | 0.28% | 36655 | 0.33% |
| intron_sense | 50725 | 0.43% | 226961 | 1.93% | 198233 | 1.7% | 393241 | 3.52% |
| miRNA | 7242379 | 61.12% | 5358725 | 45.48% | 5417857 | 46.41% | 3037591 | 27.21% |
| rRNA | 1949028 | 16.45% | 2795529 | 23.73% | 2835909 | 24.29% | 4130969 | 37% |
| repeat | 44972 | 0.38% | 159323 | 1.35% | 151950 | 1.3% | 265750 | 2.38% |
| scRNA | 14458 | 0.12% | 101387 | 0.86% | 69644 | 0.6% | 111798 | 1% |
| snRNA | 31784 | 0.27% | 75540 | 0.64% | 82245 | 0.7% | 403625 | 3.62% |
| snoRNA | 18419 | 0.16% | 95357 | 0.81% | 78921 | 0.68% | 182064 | 1.63% |
| srpRNA | 3528 | 0.03% | 10305 | 0.09% | 6895 | 0.06% | 25282 | 0.23% |
| tRNA | 127727 | 1.08% | 389850 | 3.31% | 435624 | 3.73% | 472900 | 4.24% |
| Unann* | 2289457 | 19.32% | 2024076 | 17.18% | 1974980 | 16.92% | 1529429 | 13.7% |

*unann: unannotated small RNAs
