# Supplementary material for: MiRNA expression profiles in the brains of mice infected with scrapie agents 139A, ME7 and S15
Source: Emerg Microbes Infect. 2016 Nov 9;5(11):e115–. doi: 10.1038/emi.2016.120 (PMC5148024; doi:10.1038/emi.2016.120)
Supplement: Supplementary Table 3 [file emi2016120x5.pdf]

Supplementary Table S3. The miRNAs numbers of the reads in the three scrapie infected mice and normal control

miRNA	Number of reads			
	Ctrl	139A	ME7	S15
**Up-regulated**				
mmu-miR-1291	10	33	36	27
mmu-miR-142-5p	289	704	884	667
mmu-miR-146a-5p	140	481	1384	797
mmu-miR-1940	31	101	110	858
mmu-miR-217-5p	17	50	43	70
mmu-miR-27a-3p	160	466	858	637
mmu-miR-298-5p	501	1556	1060	1545
mmu-miR-3068-3p	69	200	162	296
mmu-miR-331-3p	154	356	586	402
mmu-miR-341-3p	416	2301	3818	3339
