# Supplementary material for: MiRNA expression profiles in the brains of mice infected with scrapie agents 139A, ME7 and S15
Source: Emerg Microbes Infect. 2016 Nov 9;5(11):e115–. doi: 10.1038/emi.2016.120 (PMC5148024; doi:10.1038/emi.2016.120)
Supplement: Supplementary Table 4 [file emi2016120x6.pdf]

Supplementary Table S4. Novel miRNAs numbers of the reads in the three scrapie infected mice and normal control

miRNA	Number of reads			
	Ctrl	139A	ME7	S15
novel-mir-2	425	64	65	24
novel-mir-17	129	0	134	0
novel-mir-20	14	0	0	0
novel-mir-28	0	0	24	9
novel-mir-36	0	0	24	0
